# Efficacy Evaluation of the Mahuang-Fuzi-Xixin Decoction in Combination with Shenmai Injection for Bradyarrhythmia Treatment: A Systematic Review and Meta-Analysis

**DOI:** 10.1155/2023/7280627

**Published:** 2023-02-04

**Authors:** Zhang Zhang, Yuxin Li, Tingting Yu, Meihui Yan, Sen Li

**Affiliations:** School of Life Sciences, Beijing University of Chinese Medicine, Beijing, China

## Abstract

**Background:**

Bradyarrhythmia treatment is often not timely enough, posing a potential threat to health. It is necessary to find a strategy with stable curative effects and high safety. Mahuang-Fuzi-Xixin (MFX) decoction and Shenmai injection (SMI) are compound Chinese Patent Medicines. Recent evidence has shown that the combined use of these two drugs can effectively treat bradyarrhythmia.

**Purpose:**

To evaluate the effect of MFX decoction combined with SMI on bradyarrhythmia.

**Methods:**

PRISMA was followed as the guideline for this systematic review. RevMan 5.4 software was applied for meta-analysis.

**Results:**

Eight studies were included in the analysis, with a total of 340 patients in the intervention and control groups. The results of the meta-analysis showed that the effective rate of MFX combined with SMI treatment was higher than that of SMI alone (OR = 3.27, 95% CI (2.18, 4.89), *P* < 0.01); after treatment, MFX combined with SMI treatment and SMI alone had no significant difference in heart rate. In the subgroup of patients with an age less than 60, the effect of MFX combined with SMI treatment on the 24-hour mean heart rate was better than that of SMI alone (MD = 3.68, 95% CI (3.14, 4.22), *P* < 0.01), as did the 24-hour minimal heart rate (MD = 3.48, 95% CI (3.03, 3.93), *P* < 0.01). In addition, the effect of MFX combined with SMI treatment on the Traditional Chinese Medicine syndrome score (TCMSS) was significantly better than that of SMI alone (MD = −2.69, 95% CI (−3.10, −2.28), *P* < 0.01). In terms of safety, two adverse events were reported in the SMI combined with MFX group compared to 12 in the SMI alone group.

**Conclusions:**

MFX combined with SMI treatment is effective in treating bradyarrhythmia. However, the results were heterogeneous. The safety of MFX combined with SMI treatment should be verified and treated with caution.

## 1. Introduction

Bradyarrhythmia is a common cardiovascular disease (CVD), and every year one million people have pacemakers installed worldwide [1, 2]. Bradyarrhythmias are heartbeats fewer than 60 times per minute [2]. Generally, a slower heart rate at rest means health, which is normal among adolescents [3], but it may indicate abnormalities in the elderly [4]. Severe patients often need to install a pacemaker or use drugs such as atropine or isoproterenol to increase the heart rate [5]. In the acute phase, although these drugs can quickly restore the heart rate, they are simultaneously violent and may bring greater adverse reactions [6]. There is an urgent need to find treatment strategies with good efficacy and low toxicity.

Traditional Chinese medicine injection (TCMI) is a proprietary Chinese medicine with a long history of development [7]. People mainly use traditional Chinese medicine (TCM) orally or externally, but the emergence of TCMI can assist many clinical drugs in the treatment of severe and chronic diseases [8, 9]. Shenmai injection (SMI) is composed of *red ginseng* and *Ophiopogon japonicus*, which have been used clinically to treat bradyarrhythmia [10, 11]. Mahuang-Fuzi-Xixin (MFX) decoction, a TCM prescription, is mainly composed of *ephedra*, *asarum,* and *aconite* [12]. It is used to treat physical weakness and chills [13]. MFX decoction has been widely used to treat bradyarrhythmia and is often used in combination with other drugs [12]. For example, in the case of MFX plus atropine for treating bradyarrhythmia versus the control group using atropine alone, the efficacy after the treatment was significantly better than using atropine alone [14]. Other studies comparing the efficacy of MFX and Erxian decoction (another TCM) in the treatment of bradyarrhythmia found that compared to the control group, they can better improve the slow heart rate [15]. MFX combined with SMI has been clinically used for the treatment of bradyarrhythmias, and it has shown better efficacy than single drug. However, there is no basic research and large-scale RCT research and evaluation of the mechanism and efficacy of this strategy. Besides, the results of several RCTs are inconsistent, and the clinical evidence is insufficient. Therefore, a systematic review and meta-analysis is needed to provide clinical evidence.

This study included clinical studies in recent years, with the effective rate, 24-hour mean heart rate, 24-hour minimal heart, rate and TCM syndrome score (TCMSS) as outcome indicators, aiming to evaluate the effectiveness of the combination of MFX with SMI for treating bradyarrhythmia.

## 2. Methods

### 2.1. Databases and Search Methodology

This research method strictly adhered to Preferred Reporting Items for Systematic Reviews and Meta-analyses (PRISMA). As of November 2021, we searched the following 6 databases: the Chinese Biomedical Database (CBM), Web of Science, EMBASE, PubMed, the China National Knowledge Infrastructure Database (CNKI), and the Cochrane Library. The language of the references was limited to Chinese and English. Search terms included “Shenmai injection,” “Mahuang-Fuzi-Xixin decoction,” “bradyarrhythmia,” “bradyarrhythmias,” “bradycardia,” “Bradycardias,” “slow arrhythmia,” and “arrhythmia.” A search methodology that combined subject terms and free word lines was utilized. In addition, to avoid omissions, related references were manually searched.

### 2.2. Inclusion and Exclusion Criteria

Endnote X9 was applied to import the search results, followed by removing duplicate literature. The inclusion criteria met the following criteria: (1) compared the clinical trials of bradyarrhythmia between the SMI alone treatment group and the MFX decoction combined with SMI treatment group, (2) the control group received SMI alone, (3) the intervention group received MFX decoction combined with SMI treatment, and (4) at least one of the following results were reported: (1) efficacy, (2) 24-hour mean heart rate, (3) 24-hour minimal heart rate, and (4) Traditional Chinese Medicine Syndrome Score (TCMSS). Exclusion criteria met the following criteria: (1) incomplete data, (2) different baseline levels of patients, and (3) animal experiments, meeting reports, review comments, repeated published studies, and other nonconforming studies.

### 2.3. Data Extraction

Two experienced researchers searched and selected studies based on inclusion and exclusion criteria. If there was a disagreement in the data extraction step, the third researcher discussed and resolved the disagreement. These data were extracted: the name of the first author, the year of publication, the sample size, gender distribution, intervention measures, duration of treatment, comorbidity, outcome, and adverse events.

### 2.4. Quality Evaluation

The methodological quality of the included studies was evaluated according to the Cochrane Collaboration's tool. The risk of bias assessment mainly included five aspects: (1) the bias generated by the randomization process; (2) the deviation caused by the deviation from the expected intervention; (3) the deviation caused by the missing result data; (4) the deviation of the measurement result; and (5) the bias in the selection of the reported result. Finally, a judgment of the overall risk of bias was generated. The risk of bias was categorized into three levels: “low,” “high,” and “unclear.”

### 2.5. Data Analysis

For binary variables, the odds ratio (OR) was used to assess the effect; for continuous variables, the mean difference (MD) was used. For the above variables, the 95% confidence interval (CI) was calculated. The heterogeneity among different studies was assessed by the *χ*^2^ test and I^2^ statistic. If there was heterogeneity (I^2^ > 50% or *P* < 0.05), the random effects model was used for data analysis; otherwise, a fixed effects model was adopted. At the same time, sensitivity analysis or subgroup analysis was used to determine the source of heterogeneity. Our study did not perform a funnel plot analysis because only 8 (less than 10) studies were included. RevMan 5.4 software was employed for statistical analysis.

## 3. Results

### 3.1. Search Results

**Figure 1:**
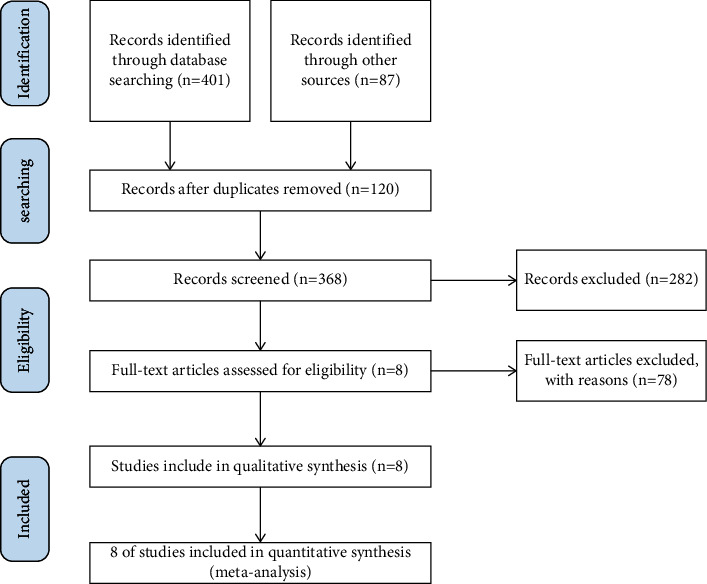
Flow diagram of study screening.

We retrieved a total of 488 studies from 6 databases. First, 120 duplicate studies were removed. Then, the title and abstract were read by experienced researchers, and 282 studies were removed because they were case reports, conference papers, or other nonconforming study types. Next, the full text was carefully checked, and 78 studies were not included in this step because their interventions or results did not meet the inclusion criteria. In the final stage, 8 studies were included [11, 16–22]. The complete screening process is shown in Figure 1.

### 3.2. Study Characteristics

Eight studies were included in the analysis, with a total of 340 patients in the intervention and control groups (170 in the intervention group and 170 in the control group). The detailed characteristics of these studies are shown in Table 1.

### 3.3. Quality Assessment of Included Studies

**Figure 2:**
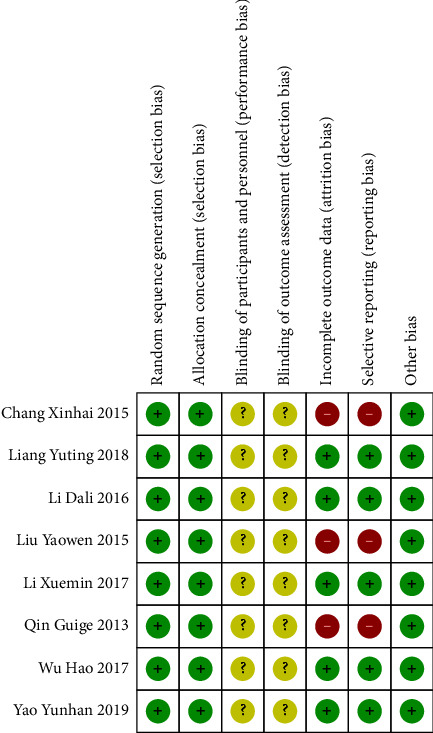
Risk of bias and summary. Note: “+,” Low risk; “?” Unclear risk; “−,” and High risk.

All studies randomly grouped patients but did not report the allocation concealment method and blinding. Two studies did not report predetermined results, nor did they report other biases. The Cochrane risk of bias results is shown in Figure 2.

### 3.4. Bias Risk Assessment

After evaluating the bias risk of included RCTs, the risk of random sequence generation and allocation concealment was found to be low. However, the blinding of participants and blinded reporting of outcome assessments remained unclear in these studies. But it was found that there were three studies with biased reporting of primary outcome and incomplete outcome data [17, 18, 20], causing high risk, while the risk of others was low (Figure 2).

### 3.5. Efficacy Assessment

#### 3.5.1. Efficacy

**Figure 3:**
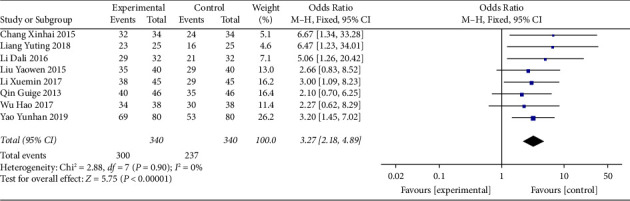
Forest plot of efficacy.

All studies reported the effective rate [11, 16–22]. A fixed effects model was used because the heterogeneity of all studies was low (I^2^ = 0). These studies had no differences in patient conditions, clinical methodology, or other characteristics. Our results indicated that MFX decoction combined with SMI was effective in the treatment of bradyarrhythmia (OR = 3.27, 95% CI (2.18, 4.89), *P* < 0.00001) (Figure 3).

#### 3.5.2. 24-Hour Mean Heart Rate

**Figure 4:**
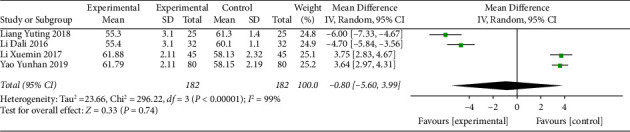
Forest plot of 24-hour mean heart rate after treatment.

**Figure 5:**
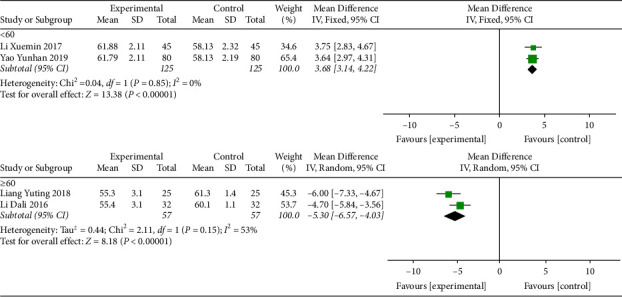
Subgroup analysis of 24-hour mean heart rate after treatment based on age.

Four studies [11, 16, 21, 22] reported the 24-hour mean heart rate. Before treatment, the 24-hour mean heart rate of patients was less than 60, and there was no difference between the intervention group and the control group (Table 1). After treatment, the 24-hour mean heart rate of patients was improved, but the heterogeneity was high (I^2^ = 99%) (Figure 4). Therefore, we conducted a subgroup analysis based on age and divided them into two subgroups, aged ≤60 and >60, to investigate the source of heterogeneity. The subgroup heterogeneity of the aged ≤60 subgroup was significantly reduced (I^2^ = 0). Although the heterogeneity of the aged >60 subgroup was reduced, it still had high heterogeneity (I^2^ = 53%) (Figure 5). It was indicated that age might be part of the sources of heterogeneity, but other sources of heterogeneity still existed. The results showed that compared to SMI treatment alone, MFX decoction combined with SMI treatment increased the 24-hour mean heart rate in patients aged less than 60 years.

#### 3.5.3. 24-Hour Minimal Heart Rate

**Figure 6:**
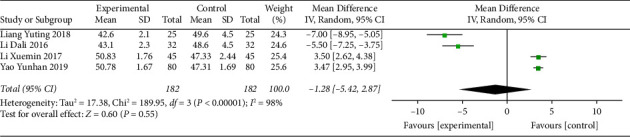
Forest plot of the 24-hour minimal heart rate after treatment.

**Figure 7:**
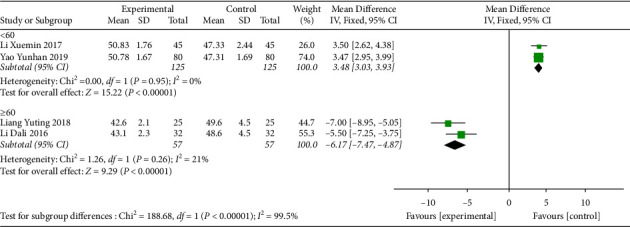
Subgroup analysis of 24-hour minimal heart rate after treatment based on age.

Heart rate, a measure of bradyarrhythmia, is often measured with a 24-hour mean heart rate and a 24-hour minimal heart rate to assess the extent of improvement of disease. Four studies [11, 16, 21, 22] reported a 24-hour minimal heart rate. Before treatment, there was no difference between the intervention group and the control group (Table 1). After treatment, the patients' 24-hour minimal heart rate was improved, but the heterogeneity was high (I^2^ = 98%) (Figure 6). Therefore, a subgroup analysis was conducted according to age and divided into two groups, aged ≤60 and >60 subgroups, to investigate the source of heterogeneity. The heterogeneity of the two subgroups was significantly reduced (aged ≤ 60 subgroup, I^2^ = 0; aged > 60 subgroup, I^2^ = 21%) (Figure 7). This indicated that age was the main source of heterogeneity, but there may be other sources of heterogeneity that have an effect on the results. The results showed that MFX combined with SMI treatment further increased the 24-hour minimal heart rate (MD = 3.48, 95% CI (3.03, 3.93), *P* < 0.00001) in patients aged less than 60 years.

### 3.6. TCMSS

**Figure 8:**
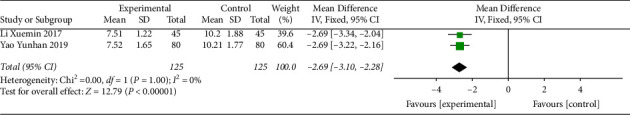
Forest plot of TCMSS after treatment.

TCMSS scores patients based on various symptoms and the lower the score, the milder the symptoms. It could be applied to determine the condition from the perspective of TCM. Two studies [11, 22] reported TCMSS. There was no difference in the TCMSS of patients before treatment (Table 1). After treatment, the patients' TCMSS decreased, and compared with SMI treatment alone, SMI combined with MFX treatment more effectively reduced the symptoms of bradyarrhythmia (MD = −2.69, 95% CI (−3.10, −2.28), *P* < 0.00001) (Figure 8). However, it should be noted that there were few studies using TCMSS as an outcome, and this conclusion needs to be treated with caution.

### 3.7. Safety

Three studies [16, 19, 21] listed the occurrence of adverse events (the total sample size was 190, and the sample size of the intervention group (MFX combined with SMI) and the control group (SMI alone) were 95 each). There were 2 cases of vomiting in the intervention group and 12 cases of adverse events in the control group (4 cases of vomiting, 2 cases of dizziness, and 6 cases of anorexia) (Table 2). One of the studies [19] did not report the specific number of adverse events. The other 5 studies [11, 17, 18, 20, 22] did not report adverse reactions. From the results, the SMI combined with MFX treatment group seemed to have higher safety, but this result needed to be treated with caution because in the studies we included, there were too few studies reporting adverse reactions.

## 4. Discussion

Through our analysis, it was found that, compared with SMI treatment alone, MFX decoction combined with SMI treatment could further improve the effective rate, 24-hour mean heart rate, and 24-hour minimal heart rate in patients less than 60 years of age and reduce the TCMSS. In terms of safety, our results showed that there were no serious adverse reactions in the included studies, and the adverse reaction rate of the intervention group was lower than that of the control group. Among the included studies, 6 studies reported treatment duration (4 weeks or 1 month), and the variety in treatment duration was small. The electrocardiogram (ECG) is an important way to continuously record heart rhythm [23]. However, the sources of these studies were quite different, and the professional level of ECG data analysts varied. This was mainly reflected in the high heterogeneity of the 24-hour mean heart rate and the 24-hour minimal heart rate after treatment. Then, we performed subgroup analysis according to age, which indicated that age may be a source of heterogeneity, and the additive effect of SMI combined with MFX on heart rate reduction exists in patients aged less than 60 years. Indeed, studies have shown that the absorption and metabolism of antiarrhythmic drugs may be different in aged patients, and thus, the administration of these drugs needs to be individualized for elderly adults [24]. For TCMSS, there was no heterogeneity, and SMI combined with MFX further decreased TCMSS compared to SMI alone. However, the number of included studies was relatively small. The accuracy of the conclusion remains to be demonstrated.

Bradyarrhythmia is a common disease [25] with symptoms of syncope, transient dizziness, fatigue, etc. [26], which cause many inconveniences to patients' lives. The clinical diagnosis of bradyarrhythmia is no longer limited to the measurement of heart rate but also includes assessment and management of the degree of bradyarrhythmia [27]. For example, after the initial medical history and physical examination, the doctor will perform a 12-lead ECG or even a Holter test on patients or laboratory tests, including thyroid function tests and electrolyte tests [5]. Bradyarrhythmia requires long-term treatment and nursing measures, which increase the financial burden on both patients and the medical system. At present, clinicians recommend alternative therapies to patients, and TCM has played an important role [12]. Indeed, the effect of MFX decoction in the treatment of bradyarrhythmia has been demonstrated [12]. Furthermore, nonrandomized clinical trials proved that MFX decoction combined with SMI can treat bradyarrhythmia. However, there are still no RCTs on the adjuvant treatment of bradyarrhythmia. Considering the efficacy of TCM treatment and the large number of nonsevere bradyarrhythmia patients, it is necessary to carry out RCT research on TCM. To provide stronger clinical evidence for MFX decoction combined with SMI treatment of bradyarrhythmia, we performed the current meta-analysis to show the efficacy of this treatment.

Blood rheology is the main factor affecting the development of arrhythmia. The increase in blood viscosity and vascular resistance in patients will cause ischemia and hypoxia in local tissues and organs [28]. Relevant pharmacological studies have shown that the mechanism of MFX in the treatment of bradyarrhythmias may involve its ability to excite *β* receptors, increase heart rate, and increase myocardial contractility. Its active ingredient, ephedra fruit polysaccharide, can improve the blood rheology of model rats and resist the formation of blood stasis [29]. There have been studies showing that SMI has a protective effect on the heart. For example, SMI can reduce mitochondrial oxidative stress and mitochondrial rupture during myocardial injury [30]. This was demonstrated in a randomized controlled study—compared with trimetazidine (TMZ) and the control group, the comprehensive treatment of SMI can improve energy metabolism in heart failure [31]. It has been reported that SMI can be combined with standard treatment to treat ischemia–reperfusion (I/R) injuries. In this process, SMI mainly alleviates the reduction of key enzymes and transporters after I/R and the utilization of metabolic substrates. Cell apoptosis was reduced, and the depletion of ATP caused by I/R was also restored [32]. The molecular mechanism of the combination treatment is not clear, but it could be evidenced from current studies on the ingredients of MFX and SMI. The active components of MFX include ephedrine and aconitine, which have been reported to increase heart rate [33]. Red ginseng, the main component of SMI, has also been reported to raise heart rate [34]. The mechanisms by which all of these components elevate heart rate involve modulation of adrenergic beta receptors (*β*-AR) and electrophysiological properties and ionic cardiomyocyte membrane channels [35]. These active ingredients jointly activate these receptors or ion channels to achieve their cumulative therapeutic effect.

### 4.1. Limitation

Our research had certain limitations. First, from the included studies, both the intervention and control groups showed high safety. However, only 3 studies reported the occurrence of adverse reactions, and the evidence was not very reliable. Therefore, we believe that the safety of the intervention cannot be fully shown. In the future, more studies need to be included to prove this hypothesis. Second, these studies had small sample sizes, and no detailed randomization or blocking strategy was provided, leading to a poor evaluation methodology. Third, the mechanism of the combination therapy was not fully revealed. Nevertheless, this is the first study to provide some evidence for the adjuvant treatment of bradyarrhythmia using combined TCMs.

## Figures and Tables

**Table 1:** Basic characteristics of included studies.

Study	Sample size	Age (years)	Sex M/F	24 h-hour mean heart rate	24 h-hour minimal heart rate	TCMSS	Intervention measures		Treatment duration	Outcomes	Adverse events
	I/C	I/C					I	C			
Yao Yunhan 2019 [11]	80/80	I: 45.63 ± 4.57	I: 45/35	I: 51.55 ± 2.69	I: 41.33 ± 3.15	I: 16.33 ± 2.15	SMI + MFX	SMI	4 w	①②③④	Not reported
		C: 45.72 ± 4.26	C: 42/38	C: 51.62 ± 2.52	C: 41.37 ± 3.28	C: 16.23 ± 2.35					
Liang Yuting 2018 [16]	25/25	I: 60.4	I: 13/12	I: 43.2 ± 4.3	I: 36.2 ± 5.0	—	SMI + MFX	SMI	—	①②③	Occurrence
		C: 62	C: 16/9	C: 42.5 ± 4.6	C: 37.4 ± 5.2						
Wu Hao 2017 [19]	38/38	I: 64 ± 6	I: 19/19	—	—	—	SMI + MFX	SMI	4 w	③	Occurrence
		C: 62 ± 6	C: 20/18								
Li Xuemin 2017 [22]	45/45	I: 42.03 ± 4.27	I: 25/20	I: 51.63 ± 3.18	I: 41.23 ± 3.57	I: 16.53 ± 2.13	SMI + MFX	SMI	—	①②③④	Not reported
		C: 41.27 ± 4.59	C: 24/21	C: 51.77 ± 3.24	C: 41.42 ± 4.06	C: 15.93 ± 3.14					
Li Dali 2016 [21]	32/32	I: 60.3 ± 3.1	I: 18/14	I: 43.2 ± 4.6<	I: 36.5 ± 5.0	-	SMI + MFX	SMI	30 d	①②③	Occurrence
		C: 61.2 ± 3.2	C: 17/15	C: 42.9 ± 4.3	C: 37.1 ± 5.1						
Liu Yaowen 2015 [17]	40/40	I/C: 42.4 ± 3.1	I/C: 43/37	—	—	—	SMI + MFX	SMI	4 w	③	Not reported
Chang Xinhai 2015 [20]	34/34	I/C: 42.4 ± 3.1	I/C: 38/30	—	—	—	SMI + MFX	SMI	4 w	③	Not reported
Qin Guige 2013 [18]	46/46	I/C: 48 ± 11.29	I/C: 51/41	—	—	—	SMI + MFX	SMI	4 w	③	Not reported

I: intervention group; C: control group; M: male; F: female; w: weeks; d: days; ① 24-hour mean heart rate; ② 24-hour minimal heart rate; ③ Efficacy; ④ TCMSS.

**Table 2:** Summary of adverse reactions.

Studies	Groups	Vomit	Dizziness	Anorexia	Adverse events rate (%)
Liang Yuting 2018	I/C	1/2	0/1	0/3	4/24.00
Li Dali 2016	I/C	1/2	0/1	0/3	3.1/18.80
Wu Hao 2017	I/C	—	—	—	10.5/13.20

I: intervention group; C: control group.

## Data Availability

The data used to support the findings of this study are included within the article.
